# Clinical Management Across CAD-RADS Categories in Patients with Intermediate CT-FFR

**DOI:** 10.3390/jcdd13090465

**Published:** 2026-09-15

**Authors:** Yanchun Chen, Wenjing Jia, Xiaoyu Ma, Yifeng Gao, Zhen Zhou, Lei Xu

**Affiliations:** Department of Radiology, Beijing Anzhen Hospital, Capital Medical University, No. 2 Anzhen Rd, Chaoyang District, Beijing 100020, China

**Keywords:** coronary computed tomography angiography, computed tomography-derived fractional flow reserve, Coronary Artery Disease Reporting and Data System, planned revascularization

## Abstract

**Background:** Interpretation of intermediate computed tomography-derived fractional flow reserve (CT-FFR) remains uncertain. We evaluated whether Coronary Artery Disease Reporting and Data System (CAD-RADS) severity modifies the association between CT-FFR and downstream management in patients with CT-FFR 0.71–0.80. **Methods:** This retrospective study included 973 symptomatic patients stratified by index-vessel CAD-RADS category and CT-FFR 0.71–0.75 versus 0.76–0.80. Planned revascularization within 90 days was the primary outcome. Multivariable logistic regression assessed CT-FFR associations and interaction with CAD-RADS; major adverse cardiac and cerebrovascular events (MACCE) were exploratory. **Results:** Planned revascularization occurred in 39.8% versus 19.3% of patients with CT-FFR 0.71–0.75 versus 0.76–0.80 (adjusted odds ratio [OR], 2.38; 95% confidence interval [CI], 1.72–3.29; *p* < 0.001). Each 0.01 decrease in CT-FFR was associated with higher odds of revascularization (adjusted OR, 1.23; 95% CI, 1.15–1.32; *p* < 0.001). The association differed by CAD-RADS category (interaction *p* = 0.011), with a more pronounced association observed in CAD-RADS 3 (adjusted OR per 0.01 decrease, 1.39; 95% CI, 1.25–1.54). During a median follow-up of 384 days, 31 patients (3.2%) experienced MACCE, with no significant adjusted association with CT-FFR. **Conclusions:** Among patients with intermediate CT-FFR, lower values were associated with a higher likelihood of planned revascularization, and the magnitude of this association differed across CAD-RADS categories.

## 1. Introduction

Coronary artery disease (CAD) remains a major contributor to cardiovascular mortality and disease burden worldwide [1]. Coronary computed tomography angiography (CCTA) has become an important first-line noninvasive test for patients with suspected chronic coronary syndromes, allowing direct visualization of coronary atherosclerosis and assessment of luminal stenosis [2]. The Coronary Artery Disease Reporting and Data System (CAD-RADS) further standardizes the anatomical severity of CAD on CCTA and provides a structured framework for subsequent testing and clinical management [3]. However, the anatomical severity of coronary stenosis does not always correspond to its hemodynamic significance. Computed tomography-derived fractional flow reserve (CT-FFR) provides lesion-specific physiological information derived from CCTA, thereby extending CCTA from primarily anatomical assessment toward an integrated evaluation of coronary anatomy and physiology [4]. Randomized trials have further demonstrated that incorporating CT-FFR into CCTA-based diagnostic pathways can influence downstream invasive coronary angiography (ICA) and revascularization decisions and improve the efficiency of subsequent clinical management [5,6].

Although 0.80 is commonly used as the clinical threshold for abnormal CT-FFR, values around this cutoff remain subject to considerable interpretive uncertainty. Expert recommendations regard CT-FFR values of approximately 0.70/0.71–0.80 as an intermediate or gray-zone range, in which reliance on a single functional threshold may be insufficient to determine the clinical significance of a coronary lesion [7,8]. This issue is particularly relevant because similar CT-FFR values may occur across different degrees of anatomic disease severity and may therefore have different implications for downstream clinical management.

Previous studies have begun to explore the integration of anatomic and functional information: incorporating CT-FFR into CAD-RADS improved therapeutic decision-making and provided additional prognostic information [9], while evidence using other functional modalities suggests that concordance between anatomic stenosis and functional impairment may help identify patients more likely to experience symptomatic improvement after revascularization [10]. However, existing studies integrating anatomic and functional assessment have predominantly evaluated broader populations with CAD, whereas evidence remains limited in patients whose CT-FFR values are confined to the intermediate range. The ADVANCE registry demonstrated that the trans-lesional CT-FFR gradient provided particularly relevant incremental information for early revascularization among patients with CT-FFR values of 0.71–0.80 [11], suggesting that clinically meaningful physiological heterogeneity persists even within this relatively narrow range. Nevertheless, it remains unclear whether the association between CT-FFR and downstream clinical management within this specific population is consistent across different degrees of CAD-RADS-defined anatomic disease severity. Therefore, this study aimed to determine whether CAD-RADS-defined anatomic disease severity modifies the association between CT-FFR and planned revascularization among patients with intermediate CT-FFR values, and to characterize downstream management across combined anatomic–functional strata. Subsequent clinical outcomes across these strata were also explored.

## 2. Methods

### 2.1. Patients

This retrospective single-center cohort study screened consecutive symptomatic patients with suspected coronary artery disease who underwent clinically indicated computed tomography-derived fractional flow reserve (CT-FFR) assessment after coronary computed tomography angiography (CCTA) at a tertiary hospital in China between 21 February and 17 November 2023. CT-FFR was clinically indicated for patients with intermediate-grade stenosis (40–90%) on CCTA and either persistent anginal symptoms or a high clinical suspicion of hemodynamically significant stenosis. Patients were eligible if at least one major epicardial coronary artery had a CT-FFR value between 0.71 and 0.80, and no coronary artery had a CT-FFR value ≤ 0.70. Patients were excluded if (1) all coronary vessels had CT-FFR values > 0.80 or at least one vessel had a CT-FFR value ≤ 0.70; (2) previous percutaneous coronary intervention (PCI) or coronary artery bypass grafting (CABG); (3) index vessel stenosis was outside the 40–90% range; (4) CCTA image quality was inadequate for subsequent analysis; (5) anomalous coronary artery origin or coronary artery fistula was present; (6) there was a history of cardiac surgery, including cardiac transplantation or valvular surgery; (7) follow-up information was unavailable. Baseline demographic characteristics, conventional cardiovascular risk factors, and other clinically relevant information were collected from the medical records. The study was approved by the institutional review board of the participating hospital and was conducted in accordance with the Declaration of Helsinki. The requirement for written informed consent was waived because of the retrospective use of deidentified clinical data.

### 2.2. CCTA Acquisition and Image Analysis

All patients underwent coronary computed tomography angiography (CCTA) using one of three CT platforms: a dual-source CT scanner (Somatom Definition Flash, Siemens Healthineers, Forchheim, Germany), a 256-section wide-detector CT scanner (Revolution CT, GE Healthcare, Waukesha, WI, USA), or a 320-detector-row CT scanner (Aquilion ONE, Canon Medical Systems, Otawara, Japan). Prospective electrocardiographic gating was used for image acquisition, with the acquisition window adjusted according to heart rate. Preprocedural pharmacologic heart-rate control was not routinely administered. Patients with marked tachycardia (>100 beats/min) or clinically relevant arrhythmias were referred for cardiologic evaluation before scanning. Examinations that did not permit reliable assessment of the coronary lumen, plaque morphology, or CT-FFR were excluded according to the predefined image-quality criteria.

Image quality was evaluated on a per-patient basis, and examinations that did not permit reliable assessment of the coronary lumen, plaque morphology, or CT-FFR were excluded. All CCTA datasets were interpreted by two readers with 6 and 7 years of experience in CCTA interpretation, respectively. Coronary stenosis severity was categorized according to the CAD-RADS 2.0 [3]. For the present study, CAD-RADS stratification was based on the stenosis severity of the index vessel corresponding to the intermediate CT-FFR measurement. When more than one vessel had a CT-FFR value within the intermediate range, the vessel corresponding to the lowest CT-FFR value was designated as the index vessel. The coronary artery calcium score (CACS) was calculated from noncontrast CT images using the Agatston method [12]. Coronary plaque morphology was qualitatively assessed on CCTA, with particular attention to high-risk plaque (HRP) characteristics. Individual HRP features, including positive remodeling, low-attenuation plaque, spotty calcification, and the napkin-ring sign, were recorded. HRP was defined as the presence of at least two of these features [13]. Interobserver agreement for HRP assessment was examined in a random sample of 70 lesions independently evaluated by a second reader, yielding good reproducibility (κ = 0.75).

### 2.3. CT-FFR Analysis

CT-FFR was derived from standard CCTA datasets using an on-site machine learning-based approach with the DEEPVESSEL FFR workstation (Keya Medical Technology Co., Ltd., Beijing, China), as previously described [14]. Major epicardial coronary arteries with a luminal diameter ≥ 2.5 mm were included in the analysis. CT-FFR values were recorded for each major coronary artery. In vessels with multiple stenoses, the CT-FFR value distal to the most severe lesion was used. Lesion-specific CT-FFR was measured approximately 20 mm distal to the lesion of interest. For patient-level analyses, the lowest CT-FFR value among all evaluated coronary arteries was selected.

### 2.4. Clinical Management and Clinical Outcomes

Post-CCTA management was determined by the treating physicians on the basis of patient symptoms, CCTA anatomy, CT-FFR findings, and other available clinical information. No prespecified management algorithm was applied, and information on the use of invasive fractional flow reserve (FFR) or instantaneous wave-free ratio (iFR) and the specific factors underlying individual revascularization decisions was not systematically available. ICA and planned revascularization performed within 90 days after CCTA were recorded. Planned revascularization was defined as PCI or CABG that formed part of the initial management strategy and was performed within 90 days. Planned revascularization involved the index vessel corresponding to the intermediate CT-FFR measurement. According to the observed downstream management pathway, patients were classified into three groups: no ICA, ICA without planned revascularization, and planned revascularization. Planned revascularization was considered the primary management outcome, whereas referral to ICA was evaluated as a secondary management outcome.

Clinical events were ascertained from medical records and follow-up data. The exploratory clinical outcome was major adverse cardiac and cerebrovascular events (MACCE), defined as a composite of all-cause death, nonfatal myocardial infarction (MI), unplanned coronary revascularization, or stroke. Nonfatal MI was adjudicated according to the Fourth Universal Definition of Myocardial Infarction [15]. Unplanned revascularization was defined as PCI or CABG that was not included in the initial planned management strategy and was subsequently performed because of recurrent ischemic symptoms or objective evidence of myocardial ischemia [16]. Stroke included ischemic or hemorrhagic cerebrovascular events established by neurological assessment and supported by imaging. For patients experiencing more than one clinical event, the first event was used for the composite MACCE analysis. Time to event was calculated from the date of the index CCTA examination to the date of the first MACCE. Patients without MACCE were censored at the date of their last available follow-up. Individual MACCE components and the composite of all-cause death or nonfatal MI were also reported separately.

### 2.5. Statistical Analysis

Continuous variables are presented as mean ± standard deviation or median with interquartile range (IQR), according to their distributions, whereas categorical variables are reported as counts and percentages. Across CAD-RADS categories 2, 3, and 4, approximately normally distributed continuous variables were compared using one-way analysis of variance and skewed continuous variables using the Kruskal–Wallis test. Categorical variables were compared using Pearson’s χ^2^ test, with Fisher’s exact test applied when expected cell counts were small. CT-FFR was evaluated using both categorical and continuous approaches. For categorical analyses, patients were divided into CT-FFR 0.71–0.75 and 0.76–0.80 groups. Comparisons of baseline characteristics between the two groups were performed as supplementary analyses.

To evaluate whether anatomic disease severity modified the association between CT-FFR and planned revascularization, an interaction term between continuous CT-FFR and CAD-RADS category was incorporated into the logistic regression model. The overall interaction was evaluated using a Wald test. CAD-RADS-specific ORs per 0.01 decrease in CT-FFR and model-adjusted probabilities of planned revascularization with 95% CIs were derived from the interaction model. As a supplementary analysis, restricted cubic spline (RCS) modeling was used to assess potential nonlinearity in the association between continuous CT-FFR and planned revascularization. Knots were placed at the 10th, 50th, and 90th percentiles of the observed CT-FFR distribution, and nonlinearity was evaluated using likelihood-ratio testing. Additional secondary analyses assessed the association between CT-FFR and ICA in the overall cohort and between CT-FFR and planned revascularization among patients who underwent ICA.

For the exploratory clinical outcomes, event frequencies and cumulative proportions were summarized according to both CT-FFR category and CAD-RADS category. MACCE-free survival was estimated using the Kaplan–Meier method and compared between CT-FFR groups using the log-rank test. Cox proportional hazards regression was used to estimate hazard ratios (HRs) and 95% CIs for the association of CT-FFR with MACCE. CT-FFR was evaluated categorically and continuously per 0.01 decrease. Given the limited number of MACCE, the adjusted Cox models were intentionally parsimonious and included age and CAD-RADS severity, with CAD-RADS entered as an ordinal covariate. The proportional hazards assumption was assessed using Schoenfeld residuals. All statistical tests were two-sided, and *p* < 0.05 was considered statistically significant. Statistical analyses were performed using Python version 3.13.5.

## 3. Results

### 3.1. Study Population and Baseline Characteristics

**Figure 1:**
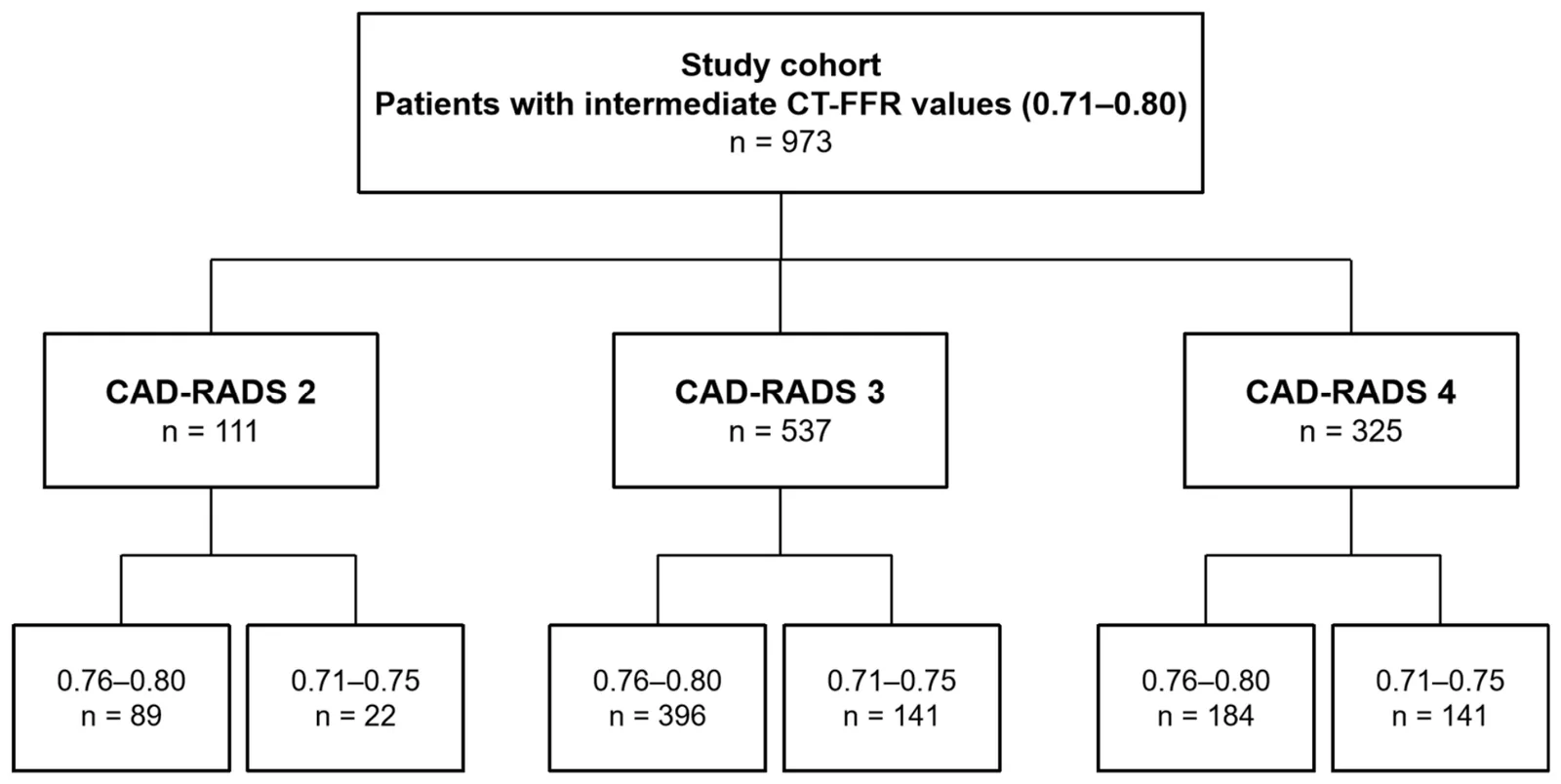
Study cohort and anatomy–physiology stratification. The final cohort included 973 patients with CT-FFR values of 0.71–0.80 and was stratified according to CAD-RADS category and CT-FFR subgroup. CT-FFR = coronary CT angiography–derived fractional flow reserve; CAD-RADS = Coronary Artery Disease Reporting and Data System.

During the study period, 52,977 patients underwent CCTA, of whom 7307 subsequently underwent clinically indicated CT-FFR analysis. After application of the prespecified eligibility and exclusion criteria, 973 patients with CT-FFR values between 0.71 and 0.80 were included in the final analysis (Figure 1). The mean age was 63.2 ± 9.4 years, and 663 patients (68.1%) were men. The mean CT-FFR was 0.76 ± 0.02; 304 patients (31.2%) had CT-FFR values of 0.71–0.75 and 669 (68.8%) had values of 0.76–0.80. According to CAD-RADS severity, 111 patients (11.4%) were classified as CAD-RADS 2, 537 (55.2%) as CAD-RADS 3, and 325 (33.4%) as CAD-RADS 4.

As shown in Table 1, CT-FFR decreased progressively with increasing CAD-RADS category (0.77 ± 0.02 for CAD-RADS 2, 0.77 ± 0.02 for CAD-RADS 3, and 0.76 ± 0.03 for CAD-RADS 4; *p* < 0.001). The proportion of patients with CT-FFR 0.71–0.75 increased from 19.8% in CAD-RADS 2 to 26.3% in CAD-RADS 3 and 43.4% in CAD-RADS 4 (*p* < 0.001). High-risk plaque and most individual high-risk plaque features were also more frequent with increasing CAD-RADS severity. Baseline characteristics according to CT-FFR category are shown in Table S1. Compared with patients with CT-FFR 0.76–0.80, those with CT-FFR 0.71–0.75 had a higher prevalence of hyperlipidemia (73.7% vs. 66.4%; *p* = 0.023), a higher CACS (median, 683.5 [IQR, 184.6–1342.1] vs. 474.7 [IQR, 169.6–975.9]; *p* < 0.001), and a greater proportion of CAD-RADS 4 disease (46.4% vs. 27.5%; overall *p* < 0.001). Other major clinical characteristics and high-risk plaque features were generally comparable between the two CT-FFR groups.

### 3.2. Downstream Management According to CAD-RADS and CT-FFR

**Figure 2:**
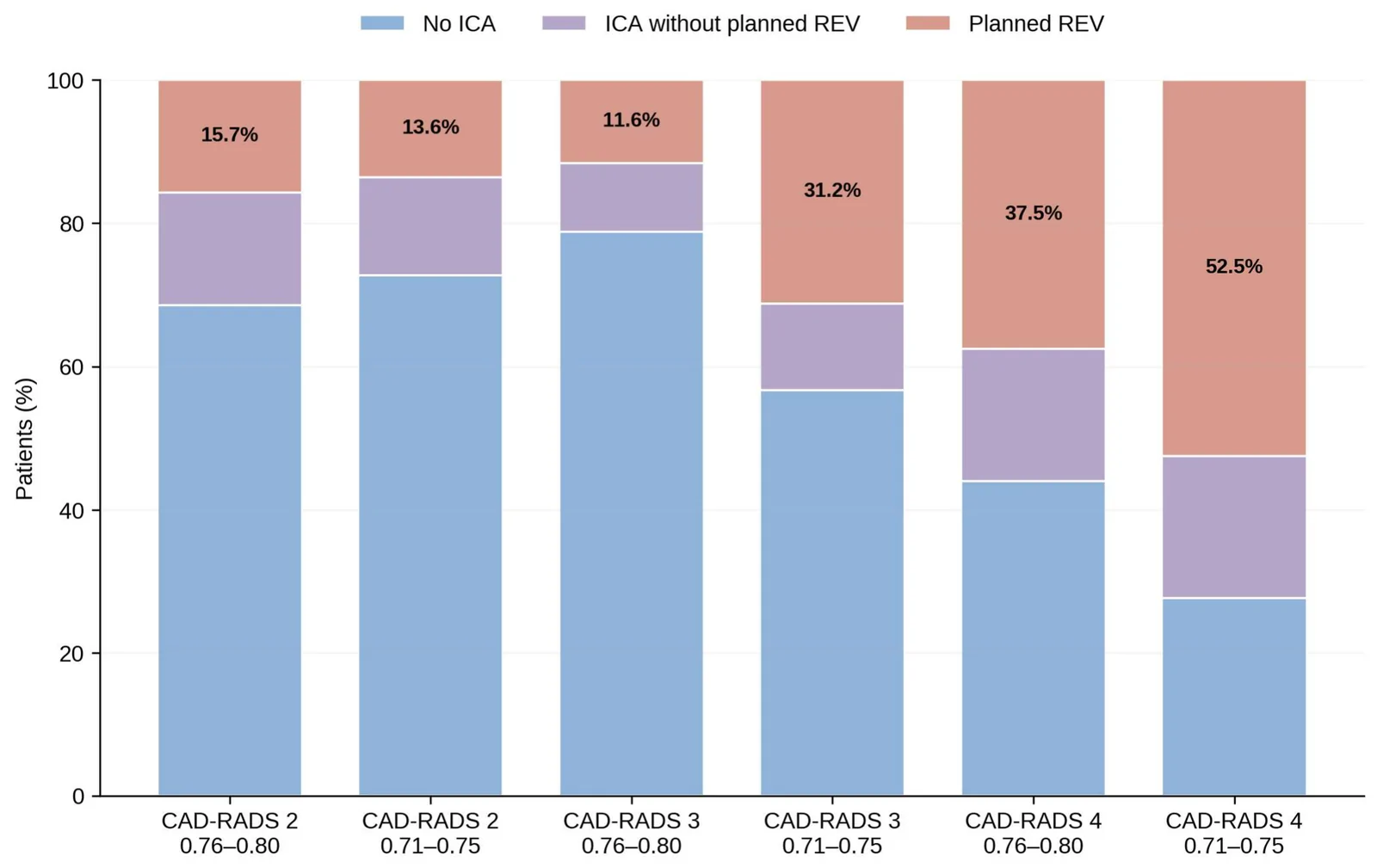
Downstream management pathways across combined CAD-RADS and CT-FFR strata. Bars show the proportions of patients who did not undergo ICA, underwent ICA without planned revascularization, or underwent planned revascularization in each CAD-RADS and CT-FFR stratum. Percentages displayed within the upper segment indicate planned revascularization rates. ICA = invasive coronary angiography; REV = revascularization; CT-FFR = coronary CT angiography–derived fractional flow reserve; CAD-RADS = Coronary Artery Disease Reporting and Data System.

Overall, 589 patients (60.5%) did not undergo ICA, 134 (13.8%) underwent ICA without planned revascularization, and 250 (25.7%) underwent planned revascularization. The distribution of these three management pathways differed across the combined CAD-RADS and CT-FFR strata (Figure 2). Among patients with CAD-RADS 2, management patterns were similar between those with CT-FFR 0.76–0.80 and 0.71–0.75, with planned revascularization rates of 15.7% and 13.6%, respectively (*p* > 0.999). In CAD-RADS 3, patients with CT-FFR 0.71–0.75 underwent ICA more frequently than those with CT-FFR 0.76–0.80 (43.3% vs. 21.2%; *p* < 0.001) and had a higher planned revascularization rate (31.2% vs. 11.6%; *p* < 0.001). A similar pattern was observed in CAD-RADS 4, in which ICA rates were 72.3% versus 56.0% (*p* = 0.002) and planned revascularization rates were 52.5% versus 37.5% (*p* = 0.007) for CT-FFR 0.71–0.75 versus 0.76–0.80, respectively. The corresponding ICA and planned revascularization rates are additionally presented as heatmaps in Figure S1.

### 3.3. Association of CT-FFR with Planned Revascularization and Effect Modification by CAD-RADS

**Figure 3:**
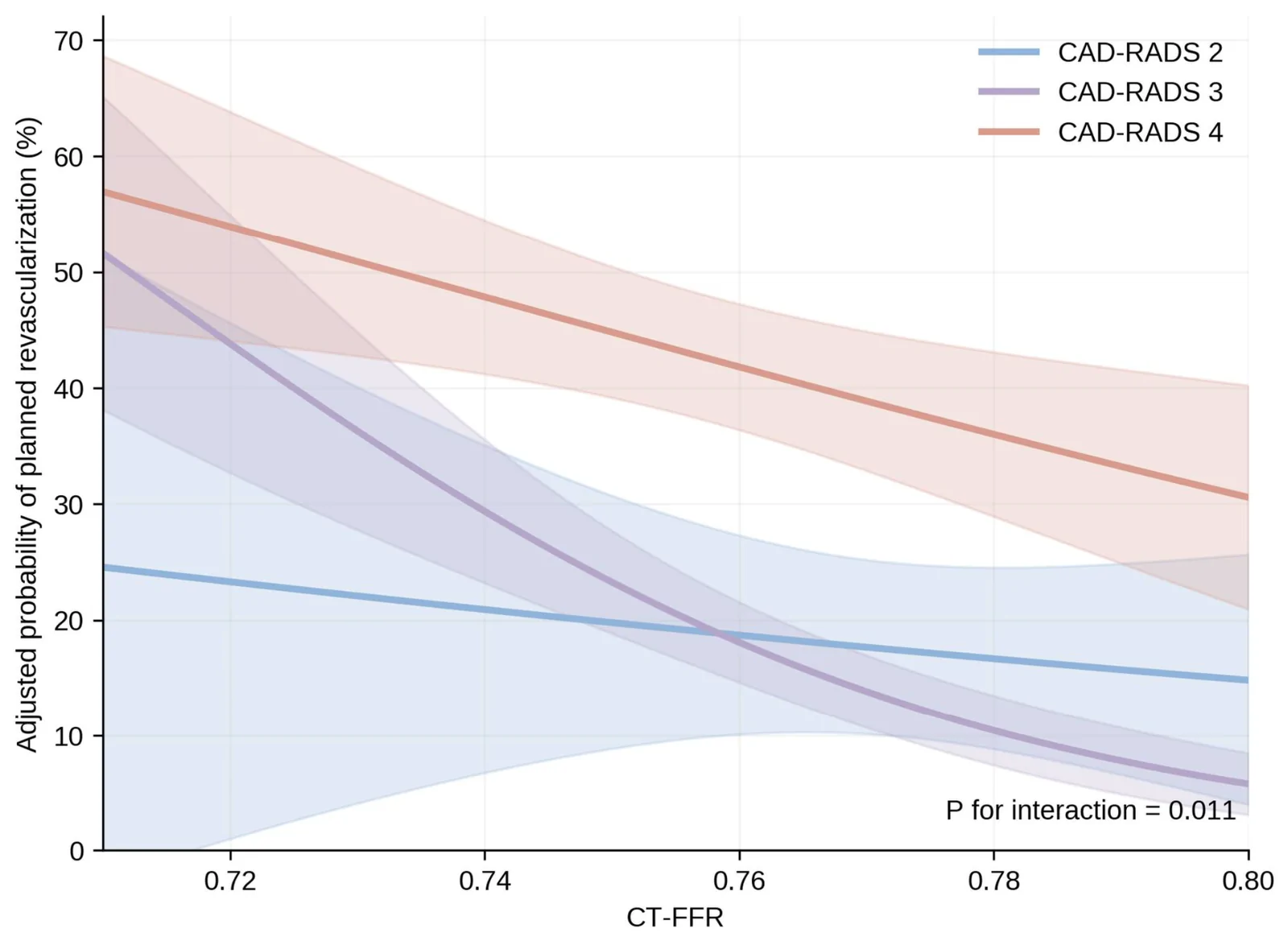
Adjusted probability of planned revascularization across continuous CT-FFR values according to CAD-RADS category. Curves show model-adjusted probabilities of planned revascularization across the CT-FFR range of 0.71–0.80 for CAD-RADS 2, 3, and 4; shaded areas represent 95% confidence intervals. *p* for CT-FFR × CAD-RADS interaction = 0.011. The model was adjusted for age, sex, hypertension, hyperlipidemia, diabetes, smoking, family history of cardiovascular disease, ln(CACS + 1), high-risk plaque, and vessel location. CT-FFR = coronary CT angiography–derived fractional flow reserve; CAD-RADS = Coronary Artery Disease Reporting and Data System; CACS = coronary artery calcium score.

Planned revascularization occurred in 121 of 304 patients (39.8%) with CT-FFR 0.71–0.75 and in 129 of 669 patients (19.3%) with CT-FFR 0.76–0.80. Compared with CT-FFR 0.76–0.80, CT-FFR 0.71–0.75 was associated with higher odds of planned revascularization in both the unadjusted analysis (OR, 2.77; 95% CI, 2.05–3.73; *p* < 0.001) and the multivariable-adjusted analysis (adjusted OR, 2.38; 95% CI, 1.72–3.29; *p* < 0.001) (Table 2). When CT-FFR was modeled continuously, each 0.01 decrease was associated with higher odds of planned revascularization (adjusted OR, 1.23; 95% CI, 1.15–1.32; *p* < 0.001). Restricted cubic spline analysis showed no evidence of significant nonlinearity (*p* for nonlinearity = 0.402; Figure S2). The association between continuous CT-FFR and planned revascularization differed according to CAD-RADS category (adjusted *p* for interaction = 0.011). The adjusted OR per 0.01 decrease in CT-FFR was 1.07 (95% CI, 0.85–1.36; *p* = 0.549) in CAD-RADS 2, 1.39 (95% CI, 1.25–1.54; *p* < 0.001) in CAD-RADS 3, and 1.14 (95% CI, 1.03–1.25; *p* = 0.008) in CAD-RADS 4 (Table 2). Consistent with these estimates, model-adjusted probability curves demonstrated the steepest increase in the probability of planned revascularization with decreasing CT-FFR among patients with CAD-RADS 3 (Figure 3).

In secondary analyses, lower CT-FFR was also independently associated with referral to ICA in the overall cohort. Compared with CT-FFR 0.76–0.80, CT-FFR 0.71–0.75 was associated with higher odds of undergoing ICA (adjusted OR, 2.29; 95% CI, 1.68–3.11; *p* < 0.001), while each 0.01 decrease in CT-FFR was associated with an adjusted OR of 1.20 (95% CI, 1.13–1.28; *p* < 0.001) (Table S2). Among the 384 patients who underwent ICA, lower CT-FFR remained associated with planned revascularization, both categorically (adjusted OR, 1.66; 95% CI, 1.05–2.62; *p* = 0.030) and continuously per 0.01 decrease (adjusted OR, 1.14; 95% CI, 1.04–1.26; *p* = 0.005).

### 3.4. Clinical Outcomes During Follow-Up

During a median follow-up of 384 days (IQR, 320–453 days), 31 patients (3.2%) experienced the MACCEs, including 14 all-cause deaths, 6 nonfatal MIs, 4 unplanned revascularizations, and 12 strokes. Individual component counts exceeded the composite count because some patients experienced more than one event. MACCE occurred in 13 of 304 patients (4.3%) with CT-FFR 0.71–0.75 and in 18 of 669 patients (2.7%) with CT-FFR 0.76–0.80 (*p* = 0.236; Table 3). Across CAD-RADS categories, no MACCE occurred among the 111 patients with CAD-RADS 2, whereas MACCE occurred in 17 of 537 patients (3.2%) with CAD-RADS 3 and 14 of 325 patients (4.3%) with CAD-RADS 4 (*p* = 0.058). No statistically significant differences were observed between the two CT-FFR groups for all-cause death, nonfatal MI, unplanned revascularization, stroke, or the composite of all-cause death or nonfatal MI.

**Figure 4:**
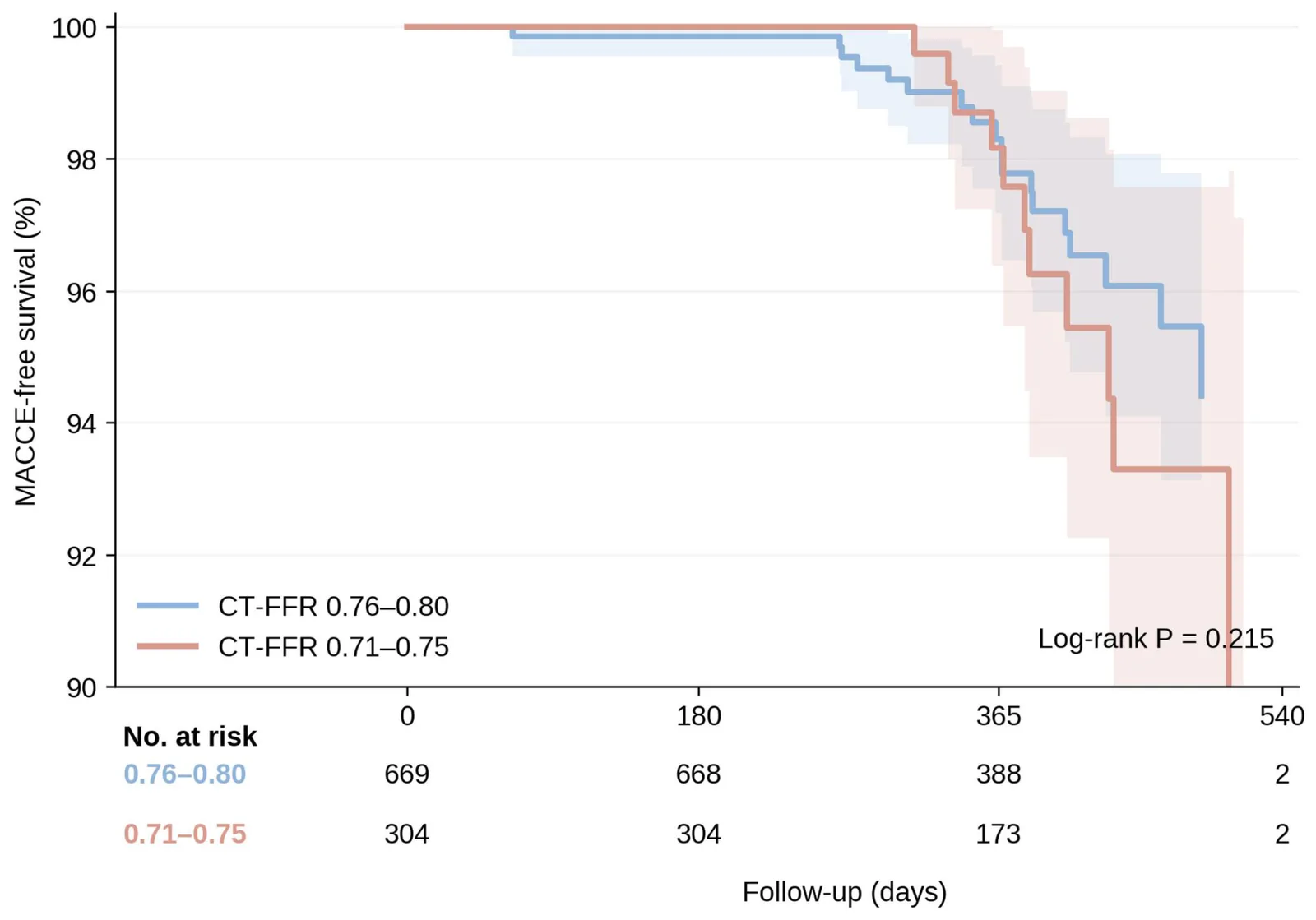
Kaplan–Meier curves for MACCE-free survival according to CT-FFR category. Kaplan–Meier curves compare MACCE-free survival between patients with CT-FFR values of 0.71–0.75 and 0.76–0.80. Shaded areas represent 95% CIs. The between-group comparison was performed using the log-rank test (*p* = 0.215). MACCE indicates major adverse cardiac and cerebrovascular events; CT-FFR, computed tomography-derived fractional flow reserve.

Kaplan–Meier analysis showed no significant difference in MACCE-free survival between the two CT-FFR groups (log-rank *p* = 0.215; Figure 4). In unadjusted Cox proportional hazards analysis, patients with CT-FFR 0.71–0.75 had a numerically higher risk of MACCE than those with CT-FFR 0.76–0.80, although the association did not reach statistical significance (HR, 1.56; 95% CI, 0.77–3.20; *p* = 0.219). When CT-FFR was analyzed continuously, each 0.01 decrease was associated with an HR for MACCE of 1.15 (95% CI, 1.00–1.33; *p* = 0.053). After adjustment for age and CAD-RADS severity, neither categorical nor continuous CT-FFR was significantly associated with MACCE (Table S3).

## 4. Discussion

In this cohort of 973 symptomatic patients with intermediate CT-FFR values of 0.71–0.80, lower CT-FFR was independently associated with a higher likelihood of planned revascularization. Each 0.01 decrease in CT-FFR was associated with a 23% increase in the adjusted odds of planned revascularization, and restricted cubic spline analysis showed no significant deviation from linearity. Importantly, the association between CT-FFR and planned revascularization varied significantly according to CAD-RADS category and was strongest in patients with CAD-RADS 3. Within this anatomically intermediate group, each 0.01 decrease in CT-FFR was associated with a 39% increase in the adjusted odds of planned revascularization, whereas the corresponding association was weaker in CAD-RADS 4 and not evident in CAD-RADS 2. Lower CT-FFR was also associated with referral to ICA and, among patients who underwent ICA, with planned revascularization. By contrast, CT-FFR was not independently associated with MACCE during follow-up. Because CT-FFR findings were available to treating physicians, these associations reflect patterns of observed downstream management and should not be interpreted as evidence that CT-FFR-guided management improves clinical outcomes.

The continuous association between CT-FFR and planned revascularization within the intermediate range is consistent with previous evidence showing that physiological information around the conventional CT-FFR threshold cannot be fully characterized by a binary classification. In the ADVANCE registry, CT-FFR substantially influenced treatment recommendations and subsequent invasive evaluation in routine clinical practice [17], while subsequent analysis showed that the trans-lesional CT-FFR gradient provided incremental information for early revascularization, particularly among patients with CT-FFR values of 0.71–0.80 [11]. The ACCURATE-CT study further demonstrated that small numerical differences within commonly defined CT-FFR gray-zone intervals may result in discordance with invasive physiological assessment and potentially different clinical classification [18]. Our findings extend this evidence from diagnostic performance and trans-lesional pressure loss to observed clinical management, showing that even when CT-FFR was confined to 0.71–0.80, the absolute value remained continuously associated with planned revascularization. This finding was also consistent with the absence of significant nonlinearity in the present spline analysis.

The significant interaction between continuous CT-FFR and CAD-RADS provides further evidence that the clinical association of physiological severity cannot be considered independently of coronary anatomy. Anatomic stenosis and physiological significance are frequently discordant, particularly among lesions of intermediate severity. The 2024 ESC guidelines emphasize physiological assessment when the hemodynamic significance of an intermediate coronary stenosis remains uncertain [2]. Studies comparing coronary anatomy with lesion-specific functional assessment have similarly demonstrated substantial anatomic–functional discordance [19,20]. A given degree of luminal narrowing may also have different physiological consequences according to the amount of myocardium subtended by the diseased vessel and other lesion-specific physiological characteristics [21,22]. These factors provide a plausible physiological basis for the variation in the association between CT-FFR and planned revascularization across CAD-RADS categories observed in the present study.

The pattern observed across CAD-RADS categories is consistent with greater uncertainty regarding functional significance in moderate anatomic stenosis. In the 2024 Chinese randomized CT-FFR trial, substantial variation in functional status was present within CAD-RADS categories, with particularly heterogeneous CT-FFR findings among patients with CAD-RADS 3 [6]. In the present study, changes in CT-FFR within the intermediate range showed a more evident association with planned revascularization in CAD-RADS 3, whereas patients with CAD-RADS 4 already had relatively high revascularization rates even when CT-FFR was 0.76–0.80. CAD-RADS 4 represents a heterogeneous spectrum of more advanced coronary anatomy. Patients in this category already had relatively high revascularization rates even when CT-FFR was 0.76–0.80. This may partly explain the weaker association between CT-FFR and revascularization in CAD-RADS 4 than in CAD-RADS 3. Of note, CACS was lower in patients with CAD-RADS 4 than in those with CAD-RADS 3. CAD-RADS is primarily determined by the severity of luminal stenosis, whereas CACS reflects the overall burden of calcified plaque; these measures therefore characterize different aspects of coronary atherosclerosis and do not necessarily increase in parallel. The higher prevalence of low-attenuation plaque in CAD-RADS 4 in our cohort further suggests that differences in plaque composition may partly contribute to this finding. No clear association was observed in CAD-RADS 2, although the estimate was less precise because relatively few patients in this subgroup had CT-FFR values of 0.71–0.75. Previous work has shown that discordance between CCTA anatomy and CT-FFR can substantially alter management decisions [23]. The present study differs from previous CT-FFR pathway studies in its level of analysis. Earlier studies primarily examined whether adding CT-FFR to CCTA altered aggregate ICA and revascularization use across broader anatomic and physiological populations [5,6,23,24,25,26], whereas the present analysis focused specifically on patients already within the intermediate CT-FFR range and demonstrated that the association between CT-FFR and planned revascularization varied according to anatomic severity.

The absence of a significant independent association between CT-FFR and MACCE should be considered in the context of the low event rate and the restricted physiological range of the present cohort. In the Chinese randomized CT-FFR trial, incorporation of CT-FFR into a CCTA-based diagnostic pathway altered the use of ICA and revascularization, whereas 1-year MACE rates did not differ significantly between strategies [6]. In contrast, studies encompassing broader CT-FFR distributions have demonstrated prognostic gradients associated with physiological severity. The ADVANCE registry reported differences in subsequent clinical outcomes according to CT-FFR status [27], and studies with longer follow-up have shown higher event rates among patients with abnormal or lower CT-FFR values [28,29], consistent with previous meta-analytic evidence [16]. In the present study, only 31 MACCE occurred, and all patients had CT-FFR values between 0.71 and 0.80. Given the small number of MACCE, the prognostic estimates were imprecise and should be considered exploratory. Accordingly, the absence of statistically significant associations does not exclude a prognostic gradient within this restricted CT-FFR range.

Several limitations should be acknowledged. First, this was a retrospective, single-center observational study, and downstream management was determined by treating physicians rather than according to a prespecified management protocol. Residual confounding could therefore have influenced the observed associations. Furthermore, detailed information regarding the use of invasive physiological assessment and the case-specific basis for revascularization decisions was not systematically available. Second, CCTA and CT-FFR findings were available to physicians when decisions regarding ICA and revascularization were made. Planned revascularization consequently represents an observed management outcome that was directly informed by the imaging parameters under investigation. The associations identified in this study do not establish a causal effect of CT-FFR on revascularization or demonstrate that a particular management strategy improves clinical outcomes. Third, the CAD-RADS 2 subgroup was relatively small, especially among patients with CT-FFR 0.71–0.75, resulting in lower precision of the effect estimate in this category. Fourth, only 31 MACCE occurred during a median follow-up of approximately 1 year, limiting statistical power for prognostic analyses and precluding more extensive multivariable or CAD-RADS-specific survival models. Sub-stratification of CAD-RADS 4 into 4A and 4B was not performed, as this patient-level classification was not directly aligned with our vessel-level analytical framework. This limitation should be considered when interpreting our findings. Finally, CT-FFR was derived using a single on-site platform, and the study population was specifically restricted to patients with CT-FFR values of 0.71–0.80 and index-vessel stenosis of 40–90%. CCTA was acquired using three different CT scanner platforms. A formal sensitivity analysis stratified by CT scanner type was not feasible due to uneven subgroup sizes and limited events across strata. Although all CT-FFR analyses were performed using the same software platform and standardized analysis workflow, scanner-specific differences in acquisition and reconstruction may have influenced CT-FFR measurements. Validation in multicenter populations using different CT-FFR algorithms and longer clinical follow-up is warranted. Emerging CT technologies may also affect the generalizability of these findings. In particular, photon-counting detector CT and evolving reconstruction techniques may alter coronary lumen and plaque characterization and, consequently, CAD-RADS classification and CT-FFR estimation. The quantitative associations observed in the present study therefore warrant further validation using PCD-CT acquisition and reconstruction protocols.

## 5. Conclusions

Among patients with intermediate CT-FFR values, the association between CT-FFR and planned revascularization varied according to CAD-RADS-defined anatomic severity and was most pronounced in patients with CAD-RADS 3. These findings highlight the interplay between anatomic and physiological information in downstream management when CT-FFR falls within the clinically uncertain range.

## Figures and Tables

**Table 1:** Baseline characteristics according to CAD-RADS category.

Characteristic	Overall	CAD-RADS 2	CAD-RADS 3	CAD-RADS 4	*p* Value
Age, years	63.2 ± 9.4	63.7 ± 8.9	63.2 ± 9.2	63.2 ± 9.9	0.878
Male, sex	663 (68.1%)	77 (69.4%)	362 (67.4%)	224 (68.9%)	0.861
Hypertension	652 (67.0%)	78 (70.3%)	369 (68.7%)	205 (63.1%)	0.173
Hyperlipidemia	668 (68.7%)	79 (71.2%)	360 (67.0%)	229 (70.5%)	0.479
Diabetes	362 (37.2%)	44 (39.6%)	213 (39.7%)	105 (32.3%)	0.082
Smoking	268 (27.5%)	31 (27.9%)	142 (26.4%)	95 (29.2%)	0.671
Family history of CVD	404 (41.5%)	55 (49.5%)	222 (41.3%)	127 (39.1%)	0.153
CT-FFR	0.76 ± 0.02	0.77 ± 0.02	0.77 ± 0.02	0.76 ± 0.03	<0.001
CT-FFR, 0.71–0.75	304 (31.2%)	22 (19.8%)	141 (26.3%)	141 (43.4%)	<0.001
CACS	534.0 [176.4–1120.5]	578.3 [242.5–1201.5]	637.0 [215.2–1160.6]	404.1 [115.8–962.6]	<0.001
Vessel location					<0.001
LAD	595 (61.2%)	50 (45.0%)	350 (65.2%)	195 (60.0%)	
LCX	147 (15.1%)	35 (31.5%)	74 (13.8%)	38 (11.7%)	
RCA	231 (23.7%)	26 (23.4%)	113 (21.0%)	92 (28.3%)	
High-risk plaque	161 (16.5%)	4 (3.6%)	92 (17.1%)	65 (20.0%)	<0.001
Positive remodeling	194 (19.9%)	8 (7.2%)	124 (23.1%)	62 (19.1%)	<0.001
Low-attenuation plaque	184 (18.9%)	2 (1.8%)	84 (15.6%)	98 (30.2%)	<0.001
Spotty calcification	113 (11.6%)	7 (6.3%)	58 (10.8%)	48 (14.8%)	0.038
Napkin-ring sign	18 (1.8%)	1 (0.9%)	2 (0.4%)	15 (4.6%)	<0.001

Unless otherwise specified, data are mean ± SD, median [IQR], or numbers of patients with percentages in parentheses, as appropriate. CAD-RADS = Coronary Artery Disease Reporting and Data System; CVD = cardiovascular disease; CT-FFR = coronary CT angiography–derived fractional flow reserve; CACS = coronary artery calcium score; LAD = left anterior descending artery; LCX = left circumflex artery; RCA = right coronary artery.

**Table 2:** Association of CT-FFR with planned revascularization overall and according to CAD-RADS category.

Population	CT-FFR Exposure	OR (95% CI)	*p* Value	Adjusted OR (95% CI)	*p* Value
Overall	0.71–0.75 vs. 0.76–0.80	2.77 (2.05–3.73)	<0.001	2.38 (1.72–3.29)	<0.001
Overall	Per 0.01 decrease	1.27 (1.19–1.35)	<0.001	1.23 (1.15–1.32)	<0.001
CAD-RADS 2	Per 0.01 decrease	1.04 (0.82–1.31)	0.744	1.07 (0.85–1.36)	0.549
CAD-RADS 3	Per 0.01 decrease	1.35 (1.22–1.50)	<0.001	1.39 (1.25–1.54)	<0.001
CAD-RADS 4	Per 0.01 decrease	1.14 (1.04–1.25)	0.005	1.14 (1.03–1.25)	0.008

Adjusted overall models included age, sex, hypertension, hyperlipidemia, diabetes mellitus, smoking status, family history of cardiovascular disease, ln(CACS + 1), high-risk plaque, vessel location, and CAD-RADS category. CAD-RADS-specific estimates were derived from the corresponding interaction models. *p* for CT-FFR × CAD-RADS interaction was 0.019 in the unadjusted model and 0.011 in the adjusted model. CT-FFR = computed tomography-derived fractional flow reserve; CAD-RADS = Coronary Artery Disease Reporting and Data System; OR = odds ratio; CI = confidence interval; CACS = coronary artery calcium score.

**Table 3:** Clinical outcomes according to CT-FFR category.

Outcome	Overall (*n* = 973)	CT-FFR 0.76–0.80 (*n* = 669)	CT-FFR 0.71–0.75 (*n* = 304)	*p* Value
MACCE	31 (3.2%)	18 (2.7%)	13 (4.3%)	0.236
All-cause death	14 (1.4%)	7 (1.0%)	7 (2.3%)	0.149
Nonfatal MI	6 (0.6%)	3 (0.4%)	3 (1.0%)	0.384
Unplanned revascularization	4 (0.4%)	3 (0.4%)	1 (0.3%)	>0.999
Stroke	12 (1.2%)	8 (1.2%)	4 (1.3%)	>0.999
All-cause death or nonfatal MI	20 (2.1%)	10 (1.5%)	10 (3.3%)	0.086

Data are numbers of patients with percentages in parentheses. MACCE was defined as all-cause death, nonfatal MI, unplanned revascularization, or stroke. Patients may contribute to more than one component event, but the composite counts each patient once. MACCE = major adverse cardiac and cerebrovascular events; MI = myocardial infarction; CT-FFR = coronary CT angiography–derived fractional flow reserve.

## Data Availability

The datasets used and/or analysed during the current study are available from the corresponding author on reasonable request.

## Supplementary Materials

The following supporting information can be downloaded at: https://www.mdpi.com/article/10.3390/jcdd13090465/s1, Table S1 Baseline characteristics according to CT-FFR category; Table S2 Secondary analyses of downstream management; Table S3 Association of CT-FFR With MACCE; Figure S1 ICA and planned revascularization rates across combined CAD-RADS and CT-FFR strata; Figure S2 Restricted cubic spline analysis of continuous CT-FFR and planned revascularization.

## Abbreviations

CAD	coronary artery disease
CCTA	coronary computed tomography angiography
CT-FFR	computed tomography-derived fractional flow reserve
CAD-RADS	Coronary Artery Disease Reporting and Data System
ICA	invasive coronary angiography
PCI	percutaneous coronary intervention
CABG	coronary artery bypass grafting
CACS	coronary artery calcium score
HRP	high-risk plaque
MACCE	major adverse cardiac and cerebrovascular events
