# Supplementary material for: Clinical Management Across CAD-RADS Categories in Patients with Intermediate CT-FFR
Source: J Cardiovasc Dev Dis. 2026 Sep 15;13(9):465. doi: 10.3390/jcdd13090465 (PMC13607401; doi:10.3390/jcdd13090465)
Supplement: Supplementary file 1 [file jcdd-13-00465-s001.zip › jcdd-4536535-supplementary.pdf]

Supplementary Materials

Table S1 Baseline characteristics according to CT-FFR category

**Characteristic**	**CT-FFR 0.76–0.80 (n=669)**	**CT-FFR 0.71–0.75 (n=304)**	**P value**
Age, years	63.4 ± 9.2	63.0 ± 9.8	0.558
Male sex	460 (68.8%)	203 (66.8%)	0.538
Hypertension	445 (66.5%)	207 (68.1%)	0.628
Hyperlipidemia	444 (66.4%)	224 (73.7%)	0.023
Diabetes	245 (36.6%)	117 (38.5%)	0.577
Smoking	178 (26.6%)	90 (29.6%)	0.332
Family history of CVD	277 (41.4%)	127 (41.8%)	0.913
CT-FFR	0.778 ± 0.013	0.735 ± 0.013	<0.001
CAD-RADS 2	89 (13.3%)	22 (7.2%)	<0.001
CAD-RADS 3	396 (59.2%)	141 (46.4%)	
CAD-RADS 4	184 (27.5%)	141 (46.4%)	
CACS	474.7 [169.6–975.9]	683.5 [184.6–1342.1]	<0.001
Vessel location			
LAD	419 (62.6%)	176 (57.9%)	0.258
LCX	101 (15.1%)	46 (15.1%)	
RCA	149 (22.3%)	82 (27.0%)	
High-risk plaque	109 (16.3%)	52 (17.1%)	0.752
Positive remodeling	138 (20.6%)	56 (18.4%)	0.425
Low-attenuation plaque	122 (18.2%)	62 (20.4%)	0.425
Spotty calcification	76 (11.4%)	37 (12.2%)	0.714
Napkin-ring sign	11 (1.6%)	7 (2.3%)	0.480

Unless otherwise specified, data are mean±SD, median [IQR], or numbers of patients with percentages in parentheses, as appropriate. CVD=cardiovascular disease; CT-FFR=coronary CT angiography–derived fractional flow reserve; CAD-RADS=Coronary Artery Disease Reporting and Data System; CACS=coronary artery calcium score; LAD=left anterior descending artery; LCX=left circumflex artery; RCA=right coronary artery.

Table S2 Secondary analyses of downstream management

Endpoint	CT-FFR	Adjusted OR (95% CI)	P value
ICA	0.71–0.75 vs. 0.76–0.80	2.29 (1.68–3.11)	<0.001
ICA	Per 0.01 decrease	1.20 (1.13–1.28)	<0.001
Planned REV among patients undergoing ICA	0.71–0.75 vs. 0.76–0.80	1.66 (1.05–2.62)	0.030
Planned REV among patients undergoing ICA	Per 0.01 decrease	1.14 (1.04–1.26)	0.005

Models were adjusted for age, sex, hypertension, hyperlipidemia, diabetes mellitus, smoking status, family history of cardiovascular disease, ln(CACS+1), high-risk plaque, vessel location, and CAD-RADS category. ICA=invasive coronary angiography; REV=revascularization; OR=odds ratio; CI=confidence interval; CT-FFR=computed tomography-derived fractional flow reserve; CACS=coronary artery calcium score.

Table S3 Association of CT-FFR With MACCE

CT-FFR	HR (95% CI)	P value	Adjusted HR (95% CI)	P value
0.71–0.75 vs. 0.76–0.80	1.56 (0.77–3.20)	0.219	1.39 (0.67–2.86)	0.374
Per 0.01 decrease in CT-FFR	1.15 (1.00–1.33)	0.053	1.12 (0.96–1.29)	0.144

HRs and 95% CIs were estimated using Cox proportional hazards regression. For categorical analysis, CT-FFR 0.76–0.80 served as the reference group. Adjusted models included age and CAD-RADS severity, with CAD-RADS entered as an ordinal variable. HR indicates hazard ratio; CI, confidence interval; CT-FFR, computed tomography-derived fractional flow reserve; CAD-RADS, Coronary Artery Disease Reporting and Data System; MACCE, major adverse cardiac and cerebrovascular events.

Figure S1 ICA and planned revascularization rates across combined CAD-RADS and CT-FFR strata

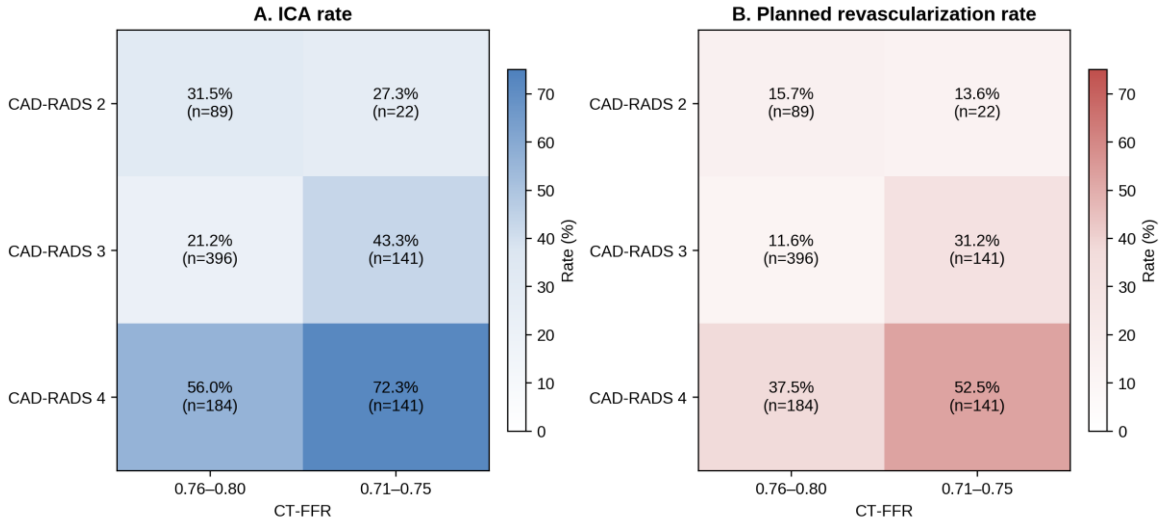

Heatmaps show the observed ICA rate (A) and planned revascularization rate (B) for each CAD-RADS and CT-FFR stratum. Each cell shows the corresponding percentage and number of patients. ICA=invasive coronary angiography; CT-FFR=coronary CT angiography–derived fractional flow reserve; CAD-RADS=Coronary Artery Disease Reporting and Data System.

Figure S2 Restricted cubic spline analysis of continuous CT-FFR and planned revascularization

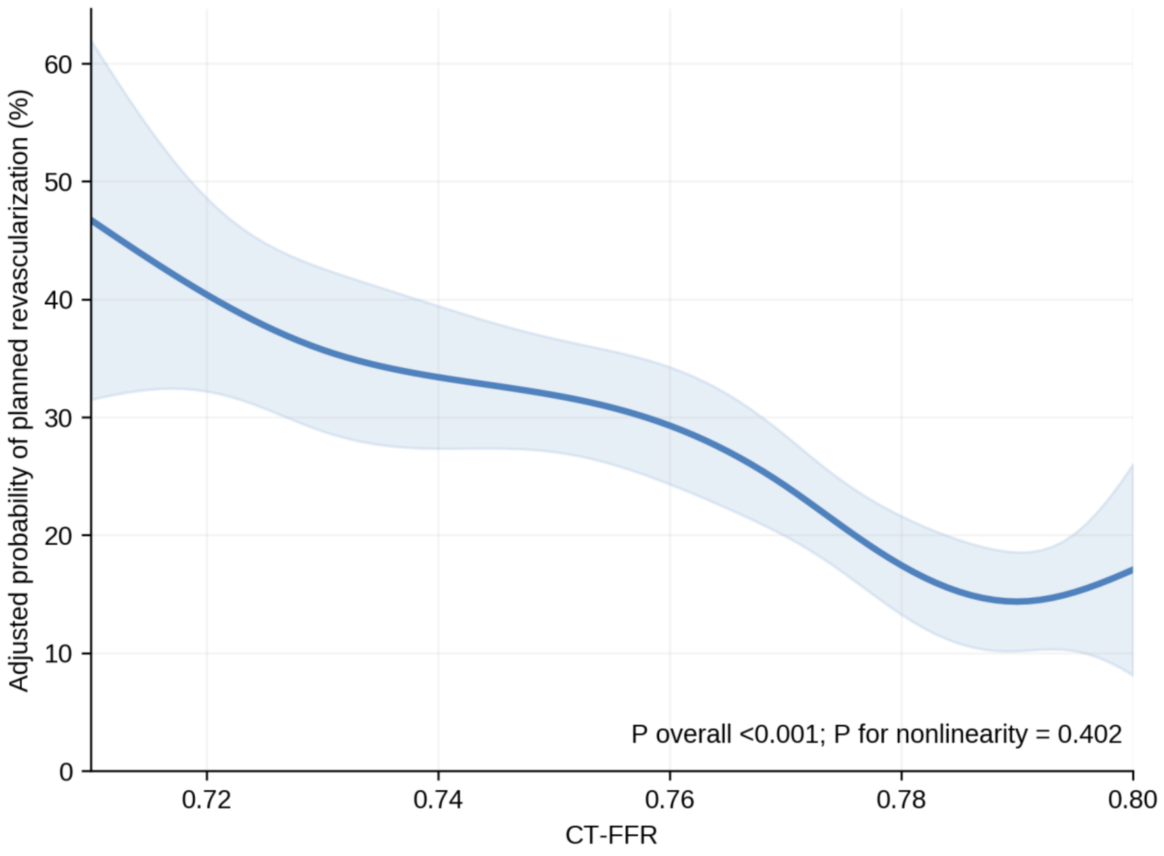

The solid line represents the model-adjusted probability of planned revascularization and the shaded area represents the 95% confidence interval. Knots were placed at the 10th, 50th, and 90th percentiles of CT-FFR (0.73, 0.77, and 0.79). P overall<0.001; P for nonlinearity=0.402. CT-FFR=coronary CT angiography–derived fractional flow reserve.
